# Comparison of the ultrasonic, Masserann, and BTR-pen techniques for removing fractured instruments with dental microscope: a CBCT-based in vitro study

**DOI:** 10.2340/aos.v85.46531

**Published:** 2026-07-20

**Authors:** Esra Balkanlıoğlu, Aliye Kamalak, Berkcan Güner

**Affiliations:** Department of Endodontics, Kahramanmaras Sutcu Imam University, Kahramanmaras, Turkey

**Keywords:** Fractured instrument removal, ultrasonic technique, Masserann kit, BTR-Pen, cone-beam computed tomography

## Abstract

**Objective:**

This in vitro study compared the efficacy, dentin loss, and outcomes of ultrasonic, Masserann, and Broken Tool Remover Pen (BTR-Pen) techniques for removing fractured endodontic instruments using cone-beam computed tomography (CBCT) analysis.

**Materials and methods:**

Ninety extracted single-rooted teeth were randomly allocated into three groups (*n* = 30). ProTaper F2 instruments were intentionally fractured in the middle third of each canal, creating 3–4 mm fragments. Removal was performed using ultrasonic tips, Masserann trephine burs, or the BTR-Pen loop system. CBCT scans quantified dentin loss. Success rates, removal times, and complications were analyzed using one-way analysis of variance, Tukey honestly significant difference, chi-square, and likelihood-ratio chi-square tests (*p* < 0.05).

**Results:**

Ultrasonic removal showed the highest success rate (90.0%), followed by BTR-Pen (86.7%) and Masserann (70.0%). Pairwise likelihood-ratio chi-square analysis showed lower success for Masserann than ultrasonic removal (*p* = 0.049). Masserann caused significantly greater canal volume increase (4.50 ± 0.50 mm³) than ultrasonic (0.75 ± 0.30 mm³) and BTR-Pen (0.93 ± 0.40 mm³) techniques (*p* < 0.001). Ultrasonic removal required the shortest time (110.0 ± 35.0 s), followed by BTR-Pen (213.4 ± 40.0 s) and Masserann (240.0 ± 50.0 s).

**Conclusions:**

Ultrasonic and BTR-Pen techniques achieved high success with better dentin preservation than Masserann. When retrieval is indicated, techniques that maximize dentin preservation should be preferred to reduce the risk of root weakening and procedural complications.

## GRAPHICAL ABSTRACT

**Figure:**
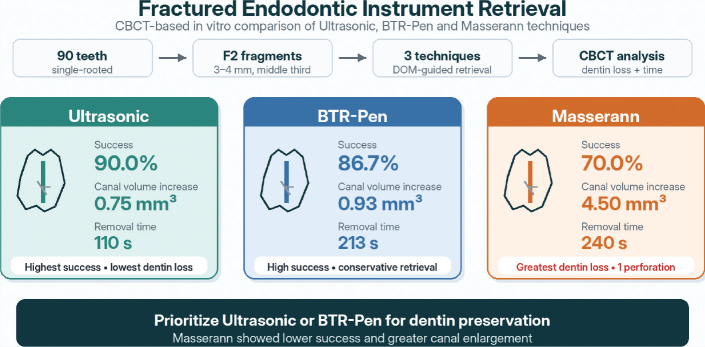

## Introduction

Instrument fracture within root canals is a well-documented complication of root canal therapy. The separation of endodontic files, usually nickel-titanium (NiTi) rotary files, can obstruct the canal and prevent proper cleaning and filling beyond the fracture site [1]. Managing a separated instrument poses a clinical dilemma: the practitioner must decide whether to attempt removal, bypass the fragment, or leave it in situ and obturate it to the level of the fragment. Removal, when feasible, is often preferred to eliminate potential impediments to therapy; however, instrument retrieval procedures carry the risk of unwanted dentin removal, canal transportation, ledge formation, or even root perforation [2].

Numerous techniques and dedicated devices have been developed to retrieve fractured intracanal instruments. Traditional methods include the use of ultrasonic tips, which vibrate around the fragment to loosen and free it, and trephine burs or tube-based systems (e.g. the Masserann kit), which drill around and engage the fragment for extraction. Ultrasonic removal under an operating microscope has become one of the most commonly employed techniques owing to its conservativeness and high success rates [3].

The Masserann kit, on the other hand, uses hollow trephine drills to circumferentially cut dentin around the metal fragment, followed by engagement with an extractor tube and screw. While the Masserann technique can be effective, especially for more coronally located fragments, its use is limited to narrow or curved canals (particularly in the apical third) because it requires a straight-line path and the removal of a considerable amount of dentin to fit the trephine around the fragment [4].

In addition, BTR‑Pen has been introduced as an innovative loop‑type retrieval system designed for use in narrow and curved canals. The BTR‑Pen employs an ultrathin, shape‑memory nitinol loop that enables secure engagement of the fragment without necessitating excessive canal enlargement [5].

Cone-beam computed tomography (CBCT) in the context of instrument removal: CBCT imaging offers a nondestructive method to evaluate internal changes in the root canal or dentin thickness before and after retrieval attempts. Using CBCT in an in vitro model allows for quantification of dentin loss (the amount of dentin removed or transported during the procedure) by superimposing pre- and postoperative scans or by directly measuring changes in canal diameter/volume at the site of fragment removal [6].

This study was designed to compare three instrument removal techniques – ultrasonic vibration, Masserann trephine, and a BTR-Pen method – in terms of their ability to successfully remove fractured NiTi instruments from root canals and the extent of dentin removal associated with each method. All procedures were conducted under a dental operating microscope to maximize visualization. Using CBCT-based measurements, we sought to objectively evaluate and compare dentin loss (i.e. internal tooth structure removed) incurred by each technique. We hypothesized that more conservative techniques (ultrasonic and BTR-Pen) would result in significantly less dentin loss than the trephine bur method, without compromising removal efficacy.

## Materials and methods

The present study was approved by the local ethical committee of Kahramanmaraş Sütçü İmam University (2024/30), and the extracted teeth were collected under informed consent in accordance with ethical guidelines.

### Sample size calculation

Assuming a medium-to-large effect size (*f* = 0.4), a significance level of 0.05 (α), and a power of 0.80 (1-β), the analysis indicated that a minimum of 66 samples (22 per group) would be sufficient. In this study, 90 samples were included (30 per group), providing an actual power of approximately 91.6%.

### Sample selection and preparation

Ninety single-rooted human premolar teeth with mature apices were selected for this study. The teeth were radiographed and examined under magnification to verify a single straight root canal (curvature < 10°). The teeth were stored in a 0.1% thymol solution at 4°C and used within 1 month of extraction.

The coronal portion of each tooth was reduced using a diamond saw under water cooling to obtain a standardized specimen length of approximately 15 mm. All canals were then instrumented using a crown-down technique to create a glide path and followed by ProTaper rotary NiTi files (Dentsply Maillefer) up to size F2 (ISO size 25, 0.08 taper) at the working length. During preparation, canals were irrigated with 2 mL of 2.5% sodium hypochlorite (NaOCl) between each file size.

To create a reproducible scenario of a fractured instrument, a new ProTaper F2 NiTi file (Dentsply) was intentionally fractured in the canal of each tooth. The intended separation point was predetermined according to the desired middle-third position within the canal, and the instrument was weakened at that level prior to fracture. The aim was to produce a fragment approximately 3–4 mm in length. After instrument separation, periapical radiographs and visual inspection under the microscope were used to verify that the fractured segment was located in the middle third of the canal and measured approximately 3–4 mm in length. Only specimens meeting these criteria were included in the study.

### Experimental groups and removal techniques

Ninety roots with fractured instruments were randomly assigned to three experimental groups of 30 samples each, corresponding to the three removal techniques to be tested.

#### Group 1 – Ultrasonic technique

Fragment removal was attempted using ultrasonic irrigation tips (ET25 ultrasonic tips, Satelec/Acteon, Merignac, France) attached to a piezoelectric ultrasonic unit. Under a dental operating microscope (Zumax Dental Microscope, Jiangsu, China) at ×16 magnification, a small staging platform was prepared around the coronal end of the fractured file using a size 2 Gates-Glidden drill as needed. This created a slightly troughed indentation in the surrounding dentin, allowing direct access to the fragment. The ultrasonic tip was then applied, energized at medium power, directly to the exposed tip of the fragment to vibrate it loosely. The ultrasound was activated in short 20-second bursts, with saline irrigation to cool and flush debris. The tip was moved circumferentially around the fragment without excessive apical pressure to gradually loosen it. If the fragment appeared loosened and visible movement was noted, micro-forceps were used to grasp and remove it from the canal. Success in this group was defined as complete removal of the fragment from the canal.

#### Group 2 – Trephine bur (Masserann) technique

Fragment removal was attempted using the Masserann kit (Micro Mega, Besançon, France), which contains hollow trephine burs and extractors of various sizes. Under the microscope, a suitably sized trephine bur (1.1 mm diameter was chosen whenever possible) was inserted into the canal to circumferentially drill around the fragment. The trephine was rotated counterclockwise with light apical pressure to cut dentin around the periphery of the broken file segment. Drilling was performed carefully and intermittently to avoid excessive heat, with irrigation used for cooling. Once the trephine had created a trough reaching the fragment’s full length (or as far as possible without perforation), the extractor tube from the kit was introduced into the space. The locking screw was then tightened to engage the fragment inside the tube, and the tube was withdrawn to pull out the fragment. Success was defined as removal of the fragment from the canal via the tube or subsequent use of ultrasound if the fragment was partially loosened.

#### Group 3 – BTR-Pen technique

Fragment removal was attempted using the BTR-Pen system (Cerkamed, Stalowa Wola, Poland), a modern fragment retrieval device that operates on a mechanical loop mechanism designed to engage and extract fractured instruments from root canals with minimal dentin removal. As with the other groups, a staging platform was first prepared using Gates-Glidden drills (typically size 2 or 3) to uncover and expose the coronal portion of the fractured fragment under a dental operating microscope (Zumax Dental Microscope, Jiangsu, China) at ×16 magnification.

The BTR-Pen’s proprietary nitinol retrieval loop (0.08–0.12 mm thickness) was inserted through the device’s applicator tip and gently maneuvered into the canal to encircle the exposed segment of the fractured NiTi file. The loop was guided circumferentially around the coronal portion of the fragment, and once engagement was visually confirmed under the microscope, the loop was gradually tightened by withdrawing the handpiece handle to cinch the fragment securely.

Controlled coronal traction was applied to extract fragments. In cases where resistance was noted, brief ultrasonic activation was delivered to the surrounding dentin (without disrupting the engaged loop) to reduce friction and aid dislodgement. The retrieval process was carefully monitored to avoid undue stress and loop breakage. Successful removal was defined as complete retrieval of the fractured file from the canal, without additional instrumentation or perforation.

To minimize variability, all procedures were performed by a single experienced operator. A dental operating microscope was used throughout all procedures. To simulate clinical conditions, the roots were embedded in silicone impression material within acrylic blocks. The total procedure time was recorded with a stopwatch from the start of the removal attempt until fragment retrieval or until the predefined maximum retrieval time had elapsed without success. The maximum active retrieval time was 4 min for the ultrasonic technique and 5 min for the Masserann and BTR-Pen techniques. Retrieval attempts were terminated earlier if further instrumentation was considered likely to cause excessive dentin removal or procedural complications.

### Cone-beam computed tomography imaging and dentin loss assessment

All teeth were scanned using CBCT before and after the instrument removal procedure to evaluate changes in the root canal and surrounding dentin. Scans were performed using a high-resolution CBCT scanner (Planmeca OY, Helsinki, Finland) with the following settings: 90 kVp, 5 mA, voxel size 75 μm, and field of view limited to the tooth (to maximize resolution). Each tooth (embedded in its block) was consistently oriented in the scanner. CBCT datasets were exported from Planmeca Romexis software (Planmeca Oy, Helsinki, Finland) and analyzed using ITK-SNAP software (version 4.2.2, University of Pennsylvania, Philadelphia, PA, USA). Volumetric dentin loss measurements were obtained by segmenting the canal space on pre- and post-operative scans and calculating the corresponding volume changes.

### Data recording and statistical analysis

**Figure 1:**
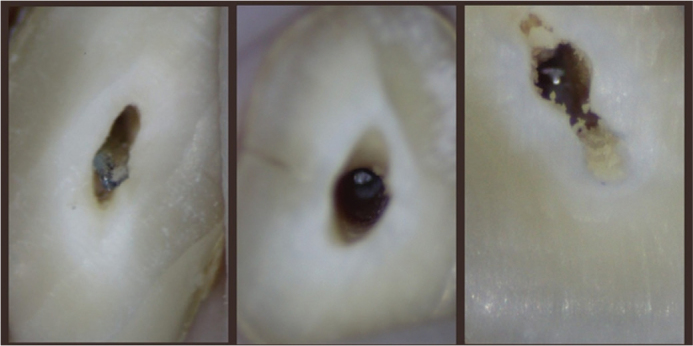
Dental operating microscope images during fractured instrument retrieval. Representative images showing retrieval procedures using (A) ultrasonic, (B) BTR-Pen, and (C) Masserann techniques.

The data were compiled and analyzed using SPSS software (v.25.0, IBM Corp., Armonk, NY, USA). Normality of the data distribution was assessed using the Shapiro-Wilk test. Since the data were normally distributed, inter-group comparisons of continuous variables (dentin loss and removal time) were performed using one-way analysis of variance (ANOVA). Post-hoc pairwise comparisons were conducted using Tukey’s honestly significant difference (HSD) test when significant differences were detected. Fragment removal success rates were compared using the chi-square test. For pairwise comparisons of categorical outcomes, likelihood-ratio chi-square tests were additionally performed. A significance level of *p* < 0.05 was set for all analyses. Representative dental operating microscope images illustrating the fractured instrument retrieval procedures are shown in Figure 1.

## Results

### Removal success and time

Table 1 summarizes the removal outcomes of each group. The ultrasonic group successfully retrieved the separated file fragments from 27 of the 30 samples, yielding a 90% success rate. In the BTR-Pen group, 26 of the 30 fragments (86.7%) were successfully removed. The difference in success rates between the ultrasonic and BTR-Pen techniques was not statistically significant (pairwise likelihood-ratio chi-square, *p* = 0.687). The Masserann trephine group had a lower success rate, with 21 of 30 samples (70.0%) successfully cleared of the fragment. Pairwise likelihood-ratio chi-square analysis indicated that the Masserann group had a significantly lower success rate than the ultrasonic group (*p* = 0.049). However, the overall Pearson chi-square comparison among the three groups did not reach statistical significance (*p* = 0.095).

**Table 1:** Comparison of success rates, procedural time, and perforation incidence among three ınstrument removal techniques.

Technique	Success rate (%)	Mean removal time (s)*	Standard deviation (s)	Perforations (*n*)
Ultrasonic	90.0	110.0	35.0	0
BTR-Pen	86.7	213.4	40.0	0
Masserann Trephine	70.0	240.0	50.0	1

BTR-Pen: Broken Tool Remover Pen.

* Procedural time represents the average time required for successful retrieval only. Failures were excluded from time calculations. Perforation data was based on CBCT and visual inspection post-procedure.

In terms of efficiency, the time required to achieve removal was the shortest for the ultrasonic technique. Ultrasonic removal, when successful, required a mean time of 110.0 ± 35.0 s. The BTR-Pen method required a longer average time, with a mean of 213.4 ± 40.0 s, reflecting the additional steps required for loop engagement and controlled traction. The Masserann group had the longest mean removal time at 240.0 ± 50.0 s for successful cases, which included the time spent drilling with the trephine and setting the extractor. One-way ANOVA showed a significant difference in removal time among the groups (*p* < 0.001). Post-hoc Tukey HSD analysis demonstrated that ultrasonic removal was significantly faster than both BTR-Pen and Masserann techniques (*p* < 0.001 for both comparisons). Although the BTR-Pen method was faster than the Masserann trephine method on average, this difference was not statistically significant (*p* = 0.080).

### Dentin loss and canal alteration (CBCT analysis)

**Figure 2:**
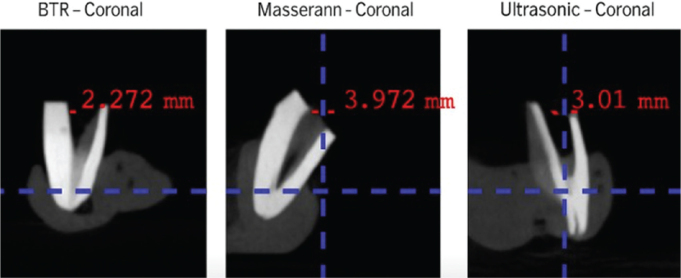
CBCT cross-sectional images of root canals after fractured instrument removal using three different techniques.

High-resolution CBCT imaging provided detailed insights into the dentin structural changes resulting from each removal technique. Figure 2 illustrates representative CBCT cross-sections for each group. The quantitative measurements of dentin loss are presented in Table 2.

**Table 2:** CBCT-based quantification of dentin loss and canal enlargement after fractured instrument removal.

Technique	Canal enlargement BL (mm)	Canal enlargement MD (mm)	Thinnest wall post-op (mm)	Canal volume increase (mm³)
Ultrasonic	0.30 ± 0.05	0.25 ± 0.04	0.85 ± 0.15	0.75 ± 0.30
BTR-Pen	0.35 ± 0.10	0.30 ± 0.08	0.70 ± 0.12	0.93 ± 0.40
Masserann Trephine	1.20 ± 0.15	1.10 ± 0.10	0.45 ± 0.10	4.50 ± 0.50

BL: buccolingual; MD: mesiodistal; BTR-Pen: Broken Tool Remover Pen.

The trephine (Masserann) technique resulted in the most substantial removal of dentin. The average increase in canal diameter at the fragment level was 1.20 ± 0.15 mm in the buccolingual direction and 1.10 ± 0.10 mm mesiodistally, far greater than the other groups (*p* < 0.001). Correspondingly, the remaining dentin thickness between the canal and external root surface was significantly reduced in trephine-treated roots. For example, the thinnest dentin wall in that region (often on the thinnest root-wall side) pre-operatively averaged 1.00 mm, but post-operatively it dropped to an average of 0.45 ± 0.10 mm, indicating roughly 50–60% loss of dentin in the most affected area. In four specimens from the Masserann group, the post-op CBCT revealed that the dentin thickness on one side was < 0.2 mm (a near perforation).

In contrast, the ultrasonic group showed minimal canal enlargement on the CBCT images. The average increase in canal diameter in the ultrasonic group was only 0.30 ± 0.05 mm buccolingually and 0.25 ± 0.04 mm mesiodistally. Many ultrasonically treated canals retained a roughly original shape, with only small notches where dentin had been selectively sanded away to free the fragment. The thinnest remaining dentin wall in the ultrasonic group post-operatively was 0.85 ± 0.15 mm on average, only marginally reduced from a pre-op value of ~1.0 mm. No ultrasonic sample had a residual wall thinner than 0.5 mm, and no perforations or cracks were detected in this group. The volumetric analysis echoed these findings: the mean increase in canal volume for the ultrasonic group was 0.75 ± 0.3 mm³, which was significantly less than the Masserann group’s mean increase of 4.50 ± 0.5 mm³ (*p* < 0.001).

The BTR-Pen group exhibited intermediate dentin loss. The average increase in canal diameter was 0.35 ± 0.10 mm buccolingually and 0.30 ± 0.08 mm mesiodistally. These values were slightly higher than those of the ultrasonic group; however, the difference was not significant for buccolingual enlargement (*p* = 0.150), whereas a minor mesiodistal difference reached statistical significance (*p* = 0.047). Compared with the Masserann technique, the BTR-Pen preserved significantly more dentin (*p* < 0.001 for both dimensions). The thinnest wall after BTR-Pen technique averaged 0.70 ± 0.12 mm, which remained above critical thickness in all cases; no BTR-Pen sample had a wall thinner than 0.3 mm, and no perforations were observed. The mean canal volume increase in the BTR-Pen group was 0.93 ± 0.40 mm³, which was significantly lower than that observed in the Masserann group (*p* < 0.001).

Statistical analysis using one-way ANOVA confirmed significant differences among the groups for all dentin loss metrics (*p* < 0.001). Post-hoc Tukey HSD comparisons showed that the Masserann group had significantly greater buccolingual enlargement, mesiodistal enlargement, and canal volume increase than both the ultrasonic and BTR-Pen groups (*p* < 0.001 for all comparisons). Between the ultrasonic and BTR-Pen groups, there was no significant difference in buccolingual widening (*p* = 0.150) or canal volume increase (*p* = 0.200), whereas a small but statistically significant difference was observed in mesiodistal widening (*p* = 0.047). No significant differences in dentin loss were found between successful and failed cases within each group, suggesting that the extent of dentin removal was mainly related to the retrieval technique rather than retrieval success.

## Discussion

The incidence of instrument fracture ranges from 0.4% to 23%, and success in endodontic treatment is heavily dependent on thorough mechanical and chemical preparation of the root canal system [7, 8]. However, the separation of endodontic instruments during canal shaping can compromise proper debridement and obturation, thereby negatively affecting overall prognosis [9, 10]. The management of separated endodontic instruments is a challenging aspect of root canal therapy, and the choice of retrieval method can significantly influence both treatment outcome and the structural integrity of the tooth. Visualization of and access to the fractured instrument are crucial factors for successful retrieval [11]. In this study, we evaluated three different removal techniques under controlled conditions and used CBCT imaging to compare how much dentin was sacrificed by each method.

Ultrasonics is widely regarded as the gold standard for the retrieval of separated instruments owing to its precision, safety, and minimally invasive nature. The dental operating microscope, together with small-diameter ultrasonic tips, allows minimally invasive root canal preparation and facilitates the safe removal of fractured instruments [9, 12].

The procedures employed during the retrieval of fractured instruments can significantly compromise the structural integrity of root canal dentin walls. In our study, one perforation occurred in the Masserann group, underscoring the invasive nature of this technique. Furthermore, Suter et al. reported that failed retrieval attempts could result in canal blockage, thereby negatively affecting the overall prognosis of endodontic therapy [13].

Our success rate for ultrasonic removal (90%) is consistent with those reported in the literature for microscope-assisted ultrasonics. Previous studies have shown that success ranges from approximately 70% to > 90% for ultrasonic retrieval, depending on the fragment location and canal curvature, and tends to be less successful in very apical fractures or if the fragment is locked in a curvature [14]. In our sample, we standardized the moderate canal curvature and fragment location to the mid-root region, likely contributing to the high success rate. The BTR-Pen technique also demonstrated a high success rate (86.7%) comparable to that of ultrasonics. This finding is consistent with the study by Dulundu and Helvacioglu-Yigit, who reported successful removal rates of 86.7% for the BTR-Pen system and 83.3% for ultrasonic retrieval, with no significant difference between the two techniques. In another recent in vitro study, Saad et al. reported an 80% success rate for BTR-Pen and a 90% success rate for the Zumax kit, with no statistically significant difference between the groups. Shajahan et al. also evaluated the BTR-Pen system in maxillary molars and reported location-dependent retrieval outcomes, indicating that fragment position remains an important determinant of success. These findings support the use of the BTR-Pen as a conservative alternative or adjunctive retrieval system, particularly when the fractured fragment is visible and can be securely engaged by the nitinol loop [5, 15, 16].

The Masserann group’s lower success rate (70%) reflects the limitations of using trephines in narrower canals. Although we used straight premolar canals, the trephine could not always completely surround the fragment without removing an excessive amount of dentin [17]. In several failures, we halted before retrieval because the risk of perforation became apparent (and indeed, one case led to perforation). Clinical reports on Masserann kits have indicated widely varying success rates. A classic study by Hülsmann reported approximately 55% success in vivo, mostly limited by canal anatomy and visibility issues [18]. Our higher in vitro success rate (70%) was likely due to the controlled setup and exclusion of extremely curved roots. These systems utilize a hollow cutting-end tube with a diameter ranging from 0.7 to 2.4 mm to expose the coronal portion of the fractured instrument during the preparation phase. However, because of the relatively large diameter of the tube compared to the root canal dimensions, significant dentin removal is often necessary to accommodate the extractor for grasping the separated fragment [17, 19].

It is generally accepted that a minimum of 0.3 mm–0.5 mm of dentin should remain around a canal after post preparation or instrumentation to maintain strength; many of our Masserann-treated samples approached this threshold, and one exceeded it, resulting in a strip perforation [20, 21]. Conversely, the ultrasonic and BTR-Pen techniques left the canal shape much closer to its original shape. The slight enlargement observed was mainly localized to troughs rather than uniform enlargement, which means that the tooth retains more structural durability [22].

Interestingly, both conservative techniques (ultrasonic and BTR-Pen) caused minor dentin loss, often on one side of the canal, which is essentially canal transportation. In our measurements, the BTR-Pen group had a slightly larger mesiodistal cut than the ultrasonic-alone group, which could be because manipulating the BTR-Pen might have worn the canal more in one direction. However, these differences are small. It is important to recognize that even ultrasonic retrieval is not entirely benign, because prolonged ultrasonic activation may abrade dentin and generate heat. Madarati et al. showed that temperature rise on the external root surface during dry ultrasonic retrieval of intracanal separated files is influenced by dentin thickness, ultrasonic tip type, power setting, and activation time, and that high power settings may produce hazardous temperature increases [23]. In a subsequent study, the same group demonstrated that active air flow significantly reduced the mean temperature rise after 120 s from 11.0°C to 4.2°C during ultrasonic removal of fractured files, supporting the use of intermittent activation and cooling strategies during retrieval procedures [24]. Therefore, in the present protocol, ultrasonic activation was applied in short bursts with saline irrigation to minimize heat generation. Budd et al. also demonstrated that coolant is important for preventing excessive heat buildup during ultrasonic procedures [25].

The main challenges of the BTR-Pen technique are its technical complexity and risk of loop snapping. We encountered a couple of instances of wire breakage. Despite these issues, the BTR-Pen method has a notable advantage: if successful, it often retrieves the fragment in a controlled manner, whereas ultrasonics might occasionally only vibrate a fragment out unexpectedly (potentially lodging it elsewhere or causing it to be swallowed, etc., if not careful). The loop essentially ‘catches’ the fragment. In clinical practice, a combination of methods is frequently employed rather than an isolated technique. For example, many endodontists initially use ultrasonics and, if they fail, resort to a Masserann or loop [26–28]. Our study isolated each method for comparison, but we recognize that a hybrid approach tailored to the case may yield the best outcome.

## Limitations

This was an in vitro study of relatively straight canals; caution is needed when extrapolating to clinical scenarios with more complex anatomies. We also standardized the fragment length and position; clinically, fragments can range widely (a long fragment in the apical third is much harder to remove than a short fragment in the coronal third). Thus, the clinical success rates could be lower than those in our controlled results. Additionally, the in vitro design did not replicate several clinical factors that may influence instrument retrieval, including saliva contamination, limited mouth opening, restricted access, and patient movement. The absence of these variables may have contributed to higher success rates than would be expected under routine clinical conditions. Moreover, all procedures were performed by a single skilled operator, and the results may vary with operator experience. Furthermore, although the use of a single experienced operator minimized inter-operator variability, differences in operator proficiency and familiarity among the evaluated techniques may have introduced operator-related bias and influenced the observed success rates and procedural outcomes.

## Conclusion

In summary, ultrasonic retrieval achieved the highest success rate (90.0%) with the lowest mean canal volume increase (0.75 ± 0.30 mm³), followed by BTR-Pen (86.7%; 0.93 ± 0.40 mm³). Masserann showed the lowest success rate (70.0%) and the greatest dentin sacrifice (4.50 ± 0.50 mm³). Clinicians are advised to prioritize minimally invasive approaches such as ultrasonics and BTR-Pen, reserving Masserann for exceptional cases with straight canal access.

## Data Availability

The data that support the findings of this study are available from the corresponding author upon reasonable request.

## Geolocation information

This in vitro study was conducted at the Department of Endodontics, Faculty of Dentistry, Kahramanmaraş Sütçü İmam University, Kahramanmaraş, Türkiye. The extracted teeth used in the study were collected in Kahramanmaraş, Türkiye.

## References

[1] Plotino G, Grande NM, Sorci E, Malagnino VA, Somma F. Influence of a brushing working motion on the fatigue life of NiTi rotary instruments. Int Endod J. 2007;40(1):45–51. 10.1111/j.1365-2591.2006.01179.x 17209832

[2] Peters OA. Current challenges and concepts in the preparation of root canal systems: a review. J Endod. 2004;30(8):559–67. 10.1097/01.DON.0000129039.59003.9D 15273636

[3] Ward JR, Parashos P, Messer HH. Evaluation of an ultrasonic technique to remove fractured rotary nickel-titanium endodontic instruments from root canals: an experimental study. J Endod. 2003;29(11):756–63. 10.1097/00004770-200311000-00017 14651285

[4] Friedman S, Stabholz A, Tamse A. Endodontic retreatment – case selection and technique. 3. Retreatment techniques. J Endod. 1990;16(11):543–9. 10.1016/S0099-2399(07)80219-6 2084213

[5] Dulundu M, Helvacioglu-Yigit D. The efficiency of the BTR-Pen system in removing different types of broken instruments from root canals and its effect on the fracture resistance of roots. Materials. 2022;15(17):5816. 10.3390/ma15175816 36079199 PMC9457077

[6] Mangal S, Mathew S, Sreenivasa Murthy BV, Nagaraja S, Dinesh K, Ramesh P. Cone-beam computed tomographic evaluation of remaining dentin thickness in bifurcated roots of maxillary first premolars after rotary instrumentation and post space preparation: an in vitro study. J Conserv Dent. 2018;21(1):63–7. 29628650 10.4103/JCD.JCD_390_16 PMC5852938

[7] Alapati SB, Brantley WA, Svec TA, Powers JM, Nusstein JM, Daehn GS. SEM observations of nickel-titanium rotary endodontic instruments that fractured during clinical Use. J Endod. 2005;31(1):40–3. 10.1097/01.DON.0000132301.87637.4A 15614004

[8] Iqbal MK, Kohli MR, Kim JS. A retrospective clinical study of incidence of root canal instrument separation in an endodontics graduate program: a PennEndo database study. J Endod. 2006;32(11):1048–52. 10.1016/j.joen.2006.03.001 17055904

[9] Fu M, Zhang Z, Hou B. Removal of broken files from root canals by using ultrasonic techniques combined with dental microscope: a retrospective analysis of treatment outcome. J Endod. 2011;37(5):619–22. 10.1016/j.joen.2011.02.016 21496659

[10] Spili P, Parashos P, Messer HH. The impact of instrument fracture on outcome of endodontic treatment. J Endod. 2005;31(12):845–50. 10.1097/01.don.0000164127.62864.7c 16306815

[11] Nevares G, Cunha RS, Zuolo ML, Bueno CE. Success rates for removing or bypassing fractured instruments: a prospective clinical study. J Endod. 2012;38(4):442–4. 10.1016/j.joen.2011.12.009 22414826

[12] Cuje J, Bargholz C, Hulsmann M. The outcome of retained instrument removal in a specialist practice. Int Endod J. 2010;43(7):545–54. 10.1111/j.1365-2591.2009.01652.x 20456518

[13] Suter B, Lussi A, Sequeira P. Probability of removing fractured instruments from root canals. Int Endod J. 2005;38(2):112–23. 15667633 10.1111/j.1365-2591.2004.00916.x

[14] Tzanetakis GN, Kontakiotis EG, Maurikou DV, Marzelou MP. Prevalence and management of instrument fracture in the postgraduate endodontic program at the Dental School of Athens: a five-year retrospective clinical study. J Endod. 2008;34(6):675–8. 10.1016/j.joen.2008.02.039 18498887

[15] Saad AY, Baraktat FT, Attia MI. Comparative analysis of the efficiency of the Broken Tool Remover-Pen versus Zumax kit in the removal of fractured NiTi files from the root canal system. An in vitro study. Saudi Endod J. 2024;14(3):348–55. 10.4103/jpbs.jpbs_1195_24

[16] Shajahan S, Dhanavel C, Raja SV, Sornamalar M, Balavaishnavi G. Comparative evaluation of the efficiency in retrieving separated reciprocating instruments using three different file retrieval systems in maxillary first molars: an in vitro study. J Pharm Bioallied Sci. 2024;16(Suppl 5):S4544–7. 40061786 10.4103/jpbs.jpbs_1195_24 PMC11888745

[17] Ruddle CJ. Nonsurgical retreatment. J Endod. 2004;30(12):827–45. 15564860 10.1097/01.don.0000145033.15701.2d

[18] Hulsmann M. Removal of fractured root canal instruments using the Canal Finder System. Dtsch Zahnarztl Z. 1990;45(4):229–32. 2257833

[19] Okiji T. Modified usage of the Masserann kit for removing intracanal broken instruments. J Endod. 2003;29(7):466–7. 12877265 10.1097/00004770-200307000-00010

[20] Hashem AA. Ultrasonic vibration: temperature rise on external root surface during broken instrument removal. J Endod. 2007;33(9):1070–3. 10.1016/j.joen.2007.06.005 17931935

[21] Saunders JL, Eleazer PD, Zhang P, Michalek S. Effect of a separated instrument on bacterial penetration of obturated root canals. J Endod. 2004;30(3):177–9. 10.1097/00004770-200403000-00012 15055438

[22] Terauchi Y, Sexton C, Bakland LK, Bogen G. Factors affecting the removal time of separated instruments. J Endod. 2021;47(8): 1245–52. 10.1016/j.joen.2021.05.003 34000326

[23] Madarati AA, Qualtrough AJ, Watts DC. Factors affecting temperature rise on the external root surface during ultrasonic retrieval of intracanal separated files. J Endod. 2008;34(9):1089–92. 10.1016/j.joen.2008.05.018 18718371

[24] Madarati AA, Qualtrough AJ, Watts DC. Efficiency of a newly designed ultrasonic unit and tips in reducing temperature rise on root surface during the removal of fractured files. J Endod. 2009;35(6):896–9. 10.1016/j.joen.2009.03.051 19482194

[25] Budd JC, Gekelman D, White JM. Temperature rise of the post and on the root surface during ultrasonic post removal. Int Endod J. 2005;38(10):705–11. 10.1111/j.1365-2591.2005.01002.x 16164684

[26] Hindlekar A, Kaur G, Kashikar R, Kotadia P. Retrieval of separated intracanal endodontic instruments: a series of four case reports. Cureus. 2023;15(3):e35694. 10.7759/cureus.35694 37012963 PMC10066733

[27] Keskin C, Keles A, Pirimoglu B, Toplu D. Endoscope-assisted retrieval of separated instruments: an ex vivo comparative study of Masserann, microsonic, and loop techniques. Proc Inst Mech Eng H. 2025;239:9544119251331711. 10.1177/09544119251331711 40219933

[28] Aminsobhani M, Hashemi N, Hamidzadeh F, Sarraf P. Broken instrument removal methods with a minireview of the literature. Case Rep Dent. 2024;2024:9665987. 10.1155/2024/9665987 38919975 PMC11196850

